# Effect of Dispersing Carbon Nanotube in Aqueous Solution by Poly-Carboxylic-Based Surfactants on Mechanical and Microstructural Properties as Cementitious Composites

**DOI:** 10.3390/ma16216880

**Published:** 2023-10-26

**Authors:** Won-Woo Kim, Jae-Heum Moon, Seung-Tae Lee

**Affiliations:** 1 KICT (Korea Institute of Civil Engineering and Building Technology), Goyang-si 10223, Republic of Korea; 2 Department of Civil Engineering, Kunsan National University, Gunsan-si 54150, Republic of Korea

**Keywords:** CNT, carbon nanotube, surfactant, cement composite, microstructure, mechanical properties

## Abstract

The development of high-performance concrete using carbon nanotubes (CNTs), which is used in various industries owing to its excellent mechanical properties, has attracted much attention, leading to ongoing research in this area. However, when mixing CNTs into cement paste, there has been limited focus on the dispersibility, and, in most cases, aqueous dispersions of CNTs used in other industrial sectors are used. Because CNTs form the structures of bundles or aggregates owing to their high aspect ratio and van der Waals force between particles, the desired dispersibility cannot be obtained when mixing CNTs in powder form with other materials. Therefore, in this study, we examined the applicability of CNT aqueous dispersions using PC-based plasticizer used in concrete. Aqueous dispersions of CNT using PC-based surfactants are prepared and their properties are compared with those of a PVP-based aqueous dispersion. To analyze the mechanical properties, the compressive strength and flexural strength are measured on the 28th day. Then, the dispersibility and microstructure are analyzed using scanning electron microscopy image analysis, thermogravimetric analysis, and BET (Brunauer–Emmett–Teller) analysis. The analysis results show the enhancement of mechanical properties due to the mixing of the CNT dispersion, and the results confirm the applicability of the proposed CNT aqueous dispersions using PC-based surfactants.

## 1. Introduction

Carbon nanotubes (CNTs) are a representative material in nanotechnology owing to their good mechanical properties and have attracted much attention from various fields. They are extensively applied in various fields because of their excellent properties such as a high Young’s modulus, a strength that is higher than that of conventional steel, and high electrical and thermal conductivity [1]. However, CNT powder forms the structures of bundles or aggregates owing to their high aspect ratio and the van der Waals force between particles, where the desired dispersibility cannot be obtained when mixing CNTs in a powder state with cement materials [2,3]. To address this problem, there has been active research involving various approaches to the dispersion of CNTs, such as their mechanical dispersion, dispersion by physical adsorption, and dispersion by chemical modification in the area of nanofluid preparation methods [4]. Representative methods for enhancing the water solubility of CNTs include chemical modification through acid treatment and dispersion using a physical method. In particular, there has been active research on the water solubilization of CNTs using polyvinyl pyrrolidone (PVP)-based polymers, which are a type of polymer compound, as surfactants [5,6], as these are commonly used in other industries. Another method is dispersion using isopropanol. Although effective in dispersing CNTs, when dispersing using isopropanol [7,8], there is a process in which isopropanol needs to be replaced again, and so it was not suitable for manufacturing CNT aqueous solutions for construction purposes that require mass production.

In the construction industry, studies have been conducted globally to realize the improvements in the physical properties of concrete using CNTs or to enhance the electromagnetic shielding effectiveness of cement composites using the high electrical conductivity of CNTs, in addition to exploiting the mechanical performance of CNTs [9,10,11]. However, in most of these studies, CNTs are applied in powder form for mixing with concrete, or aqueous dispersions of CNTs prepared for use in other industrial sectors are used without appropriate modifications. In addition, in the OPC–CNT composites proposed in existing research, there have been few analyses of the effect of the dispersion state of CNTs in concrete and surfactants of CNT aqueous dispersions on the improvement of the mechanical properties of the concrete.

In this study, a bead mill and nano-disperser were used as tools for the physical dispersion of CNTs to prepare CNT aqueous dispersions specialized for cement mixing in concrete. A poly-carboxylic (PC)-based superplasticizer used in concrete mixing was used as the surfactant for aqueous dispersion preparation, and as a control group for comparison, aqueous dispersions prepared with PVP, which is a high-molecular-weight polymer used in other industries, were used. Several researchers [12,13,14,15] have proposed that PC-based superplasticizers are suitable for dispersing CNTs as surfactants. In particular, Vesmawala et al. [11] noted that polycarboxylate-based surfactant has double dispersing capacity to disperse CNT particles and cement particles. And, when Silica fume is used, the dispersion effect is better, and the use of cement composites helps to increase the mechanical properties [16,17,18,19].

For the cement paste with the application of each surfactant of CNT aqueous dispersion, the Vicat needle test, which obtains the penetration resistance measurements and thermogravimetric analysis (TGA), was performed to analyze the effect on the hydration reaction, and the mechanical properties were analyzed by measuring the compressive strength and flexural strength. In addition, the microstructure was analyzed using scanning electron microscopy (SEM) images and the Brunauer–Emmett–Teller (BET) test results to investigate the effect on the mechanical properties and the causes of the changes in the properties.

## 2. Experimental Design and Methods

### 2.1. Preparation of CNT Aqueous Dispersions

In this study, two types of surfactants were used for the preparation of CNT aqueous dispersions: PC-based surfactants, which are widely used as superplasticizers for high-performance concrete, and PVP-based surfactants, which are commonly used in other industrial sectors.

**Figure 1:**
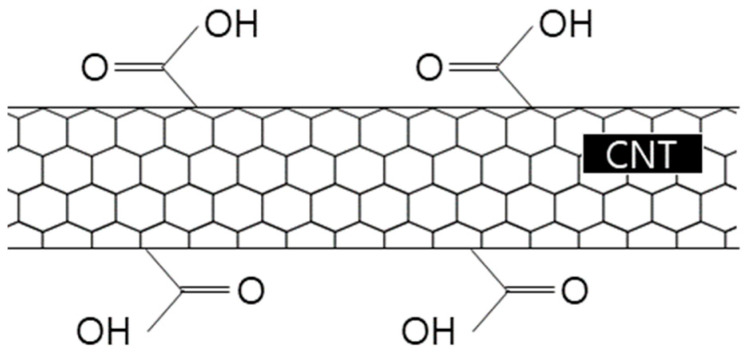
Structure of PC-based surfactants binding to CNT [11].

PC-based surfactants are considered to be effective for use in aqueous dispersions because carboxyl groups (COOH^−^) are adsorbed on the surface of CNTs, causing hydroxy (OH^−^) groups to have an affinity to water. Figure 1 illustrates the structure of the PC-based surfactants binding to CNTs [20,21].

**Figure 2:**
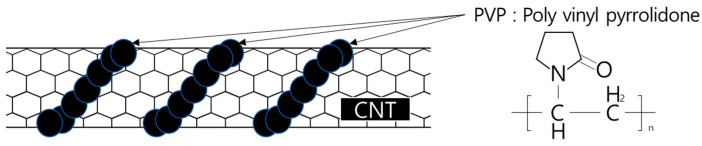
Structure of PVP-based surfactants binding to CNT [11].

In the case of PVP-based surfactants, because each molecular unit of pyrrolidone is a long-chain polymer chemically bonded to the main chain of polyethylene, they wrap around the CNTs, as shown in Figure 2 which is a schematic illustration. As shown in Figure 2, because PVP wraps around CNTs in the form of chains, the molecular stability of the PVP-based aqueous dispersions is determined to be superior to that of the PC-based surfactants based on chemical adsorption [22,23].

**Figure 3:**
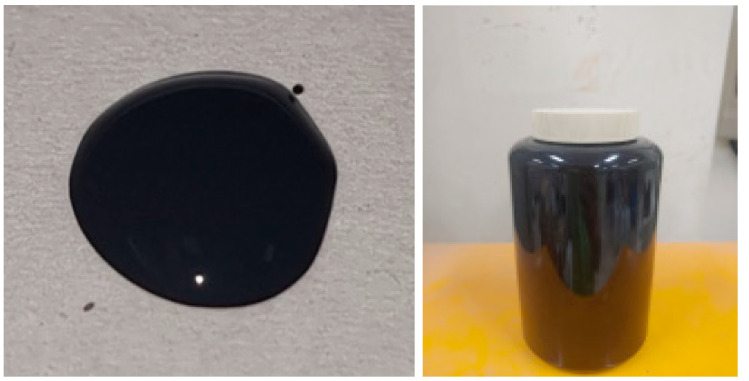
CNT aqueous dispersion.

As for the amount of surfactant added to the CNT dispersion, the amount minimized the mechanical properties and cement hydration delay effect of PC surfactant, and for PC-based surfactants, 20% of the solid aqueous dispersion was used, and the applied ratio of water/CNT/dispersant was 95:1:4. For PVP dispersants, the applied ratio of water/CNT/dispersant was 98.5:1:0.5. In this case, triple-distilled water (ultrapure water) was used as the water (Figure 3). The amount of PC used as the CNT surfactant was the amount suggested by Kim [24]. The characteristics of CNT are shown in Table 1.

In order to use it as a construction material, bulk production is required. For sonication, it is possible to produce it at the laboratory scale, but it is not appropriate for bulk production. Therefore, the method used for the bulk production of CNT aqueous solution using PVP by other industrial sectors was used.

Bead milling was performed for 100 passes, and a nano-disperser was applied three times to prepare the CNT aqueous dispersions. In bead milling, beads between rotors rotate at a high speed and dispersion is performed by collisions between particles, used to disperse them homogeneously, and the nano-disperser, with which high-pressure fluids are accelerated and dispersed by shear forces [25,26]. When the bead mill and nano-disperser were used for the preparation of aqueous dispersions, the viscosity of the aqueous solution increased rapidly and then decreased after reaching the peak value. In this study, the point at which the viscosity decreased and then showed no change after reaching the peak value was determined to be the saturated state for the solution. Based on this state, it was determined that bead milling should be performed for 100 passes, and the nano-disperser was applied three times for the experiment.

When selecting the methods of physical dispersion, for dispersion methods when dispersing a bulk scale mass in a short time using a homo mixer or ultrasonication, CNTs may be physically damaged using ultrasonic waves [27]. Thus, bead mills and nano-dispersers were used to maximize the effect of dispersion by generating a strong shear flow for the direct dispersion of CNTs in an aqueous solution [28,29].

### 2.2. Materials and Methods of Cement Paste Mixing

Table 2 lists the mix proportions of the cement paste employed in this study. The binder used was Ordinary Portland Cement (OPC) and silica fume of Elkem’s 940U (Table 3). It was used to confirm the existing research results that silica fume has an effect on the dispersion of CNTs. The water–binder ratio (w/b ratio) was set to low water–binder; considering the high price of CNT, the water–binder ratio was 0.3, determined for the development of high-strength concrete. And, to keep the w/b ratio constant, the amount of water contained in the CNT aqueous dispersion was excluded from the water in the amount of mixing water. The chemical admixture was determined based on the flow at 200 mm considering the mixing and formability to minimize the effect on the cement paste. For the preparation of cement paste, mixing was performed using a paddle mixer according to ASTM C 305 [30], and the CNT aqueous dispersion was added by mixing with the mixing water in advance.

## 3. Experimental Method

### 3.1. Vicat Needle Test for Penetration Resistance Measurement

The Vicat needle test method was used to measure the time required for the cement paste to be set (ASTM C 191 [31]). The initial and final setting times were derived by measuring the progress of the cement paste setting time based on the penetration depth of the Vicat needle. VICAMATIC2 of CONTROLS was used as the equipment to perform automated measurements during the Vicat needle test.

### 3.2. Thermogravimetric Analysis (TGA)

An instrumental analysis was performed to measure the TGA of the cement paste. The specimens were crushed to an appropriate size after curing for 28 days. And, the specimens were stored by immersing them in acetone to stop hydration. Then, they were heated to remove impurities such as moisture. SDT650 (TA Instruments, New Castle, DE, USA) was used as the analysis instrument, and the analysis was performed in a nitrogen atmosphere. The temperature was then heated to 1000 °C at a constant rate of 10 °C/min.

### 3.3. Measurement of Compressive Strength and Flexural Strength

The compressive strength for each mix proportion of the cement paste was measured using a 300-ton universal testing machine (UTM) after dry-curing for 1 d, and then cured in water with lime until the 28th day. The ALPHA 3-3000S Form Test was used for the UTM, and a 50 × 50 × 50 mm specimen was used for the compressive strength measurement according to the guidelines in ASTM C 109 [32], and the average value of the measurements of the three specimens was used for the analysis.

The flexural strength was measured using a 300 kN UTM after the specimens were water-cured for 28 d. The analysis instrument that was used was AG-300KMX of SHIMADZU, and the test specimens for flexural strength were fabricated with dimensions of 40 × 40 × 160 mm with ASTM C 348 [33], and the analysis was performed using the average value of three specimens’ measurements by employing a three-point load flexural test.

### 3.4. SEM Analysis

To examine the CNT morphology and dispersion, SEM images for the 28th day cement paste were obtained, and Hitachi’s SU8220 (Tokyo, Japan) (high-resolution field-emission scanning electron microscope) was used as the SEM analysis instrument. After the compressive strength test, the crushed paste specimens were placed in the sample holder and stored with the hydration stopped, and samples of a size appropriate for conducting SEM analysis were prepared.

### 3.5. BET Analysis

To investigate the correlation between the strength, specific surface area, pore size, and pore volume according to OPC–CNT composites, MicrotracBEL’s BELSORP-max II was used. For the instrumental analysis, the specimens were crushed to an appropriate size after curing for 28 days. And the specimens were stored by immersion in acetone to stop hydration. Then, they were heated to remove impurities such as moisture. BELPREP-VAC II was used for the pre-treatment of the samples, and after applying a vacuum to the sample cell containing the samples, small amounts of nitrogen gas were added, and the degrees of gas adsorption and desorption for pressure were measured using a pressure gauge. The measurement values were substituted in the BET theory to obtain the final result values for the BET analysis.

## 4. Experimental Results and Analysis

### 4.1. Results of Vicat Needle Test

**Figure 4:**
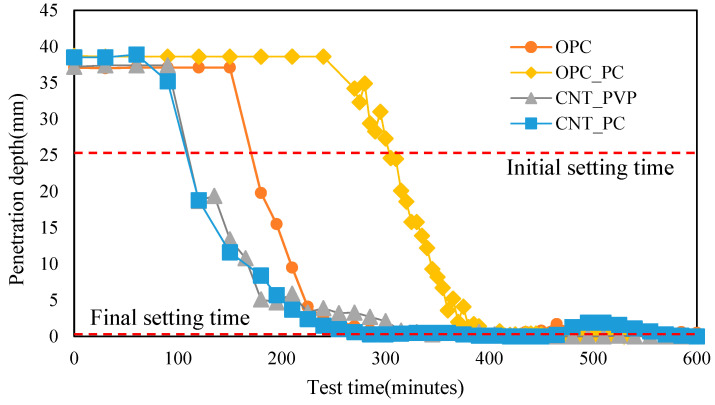
Vicat needle test for penetration resistance of cement composites.

The results of the Vicat needle test are shown in Figure 4. The setting time of the cement paste was determined according to ASTM C 191 [31], and in the case of OPC, the initial setting time measured 174 min after mixing. For the samples containing the CNT aqueous dispersion with PVP surfactants, the initial setting time measured at 112 min, and when PC-based surfactants were used as a dispersant, the initial setting time was measured at 109 min. When CNT aqueous dispersions were mixed, the setting time decreased by approximately 1 h compared to that of OPC, regardless of the type of surfactant used. In addition, the final setting time was measured at similar values between 280 and 300 min for all cases. Generally, PC surfactant delayed cement hydration. As shown in Figure 4, this caused a delay of about 1 h compared to OPC when the same amount used for CNT dispersion with PC surfactant was used. However, when CNT dispersed with PC surfactant was incorporated, the final setting time was similar to that of the OPC final setting time, and the initial setting time was short.

**Figure 5:**
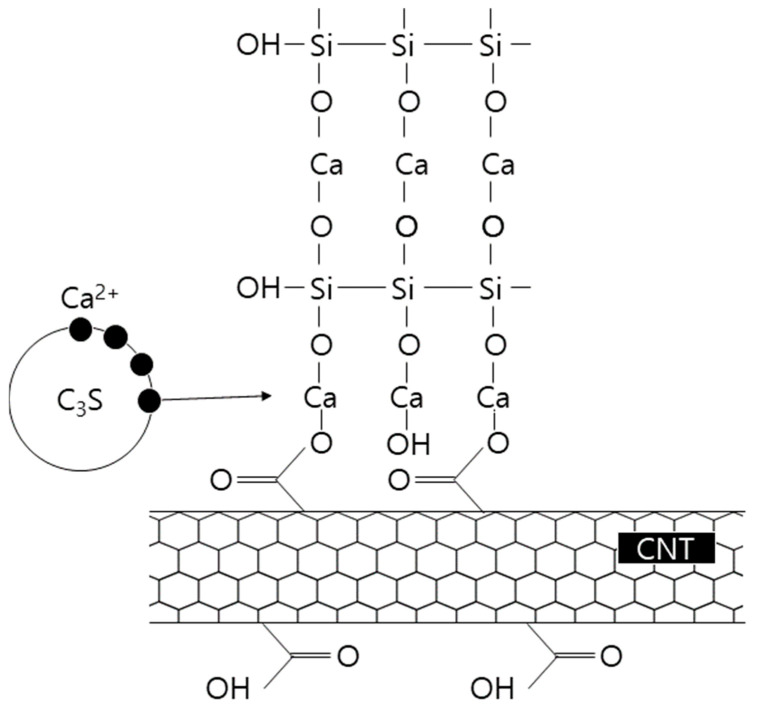
Schematic of CNT-PC-CSH formation.

It is believed that because of the surface potential of CNTs, Ca^2+^ ions of C_3_S, which are mostly present in cement particles, move close to the CNTs and have a role as a nucleation seed for the formation of cement hydrates so that calcium silicate hydrates (C-S-H) can be formed in an accelerated initial cement hydrate reaction (Figure 5). In particular, when PC-based surfactants are used, OH^−^ is adsorbed on the surface of CNTs by COOH^−^ groups, forming C-S-H, as shown in Figure 5, which is considered to accelerate the initial hydrate reaction. However, the final setting times gradually converged to similar values. This indicates that the mixing of CNTs acts as a bridge that promotes C-S-H formation, rather than forming chemical bonds, in the overall process of C-S-H formation, which is believed to have not affected the total amount of C-S-H hydrate formation. This indicates that CNTs have the role of a nucleation seed that promotes C-S-H rather than forming a chemical reaction in the process.

### 4.2. Results of Thermogravimetric Analysis

**Figure 6:**
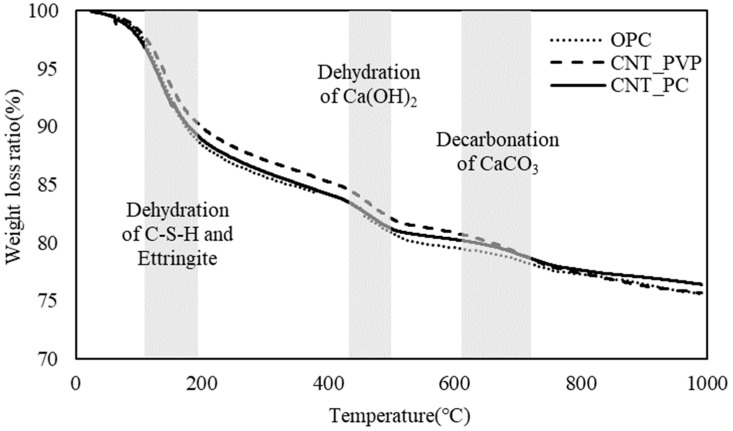
Result of thermogravimetric analysis of cement composites.

Thermogravimetric analysis (TGA) was conducted to OPC–CNT composites of 28 days, and it increased at a constant rate of 10 °C/min up to 1000 °C. The weight loss curve obtained from the analysis is shown in Figure 6. The results confirmed that there was no significant difference between OPC and OPC–CNT composites. The weight loss ratio obtained by the dehydration of C-S-H and ettringite was in the range of 80–160 °C, and the weight loss obtained by the dehydration of Ca(OH)_2_ was observed at approximately 450 °C. At 665–800 °C, weight loss due to the decomposition of CaCO_3_ into CaO and CO_2_ (decarbonation) was observed. In the overall results, the weight loss ratio due to the dehydration of C-S-H hydrate was the largest, and this result was similar to the values reported in previous studies in the weight loss curve of cement [29,34]. Therefore, as with the results of the Vicat needle test, the TGA results also showed that CNTs do not contribute to the formation of a chemical reaction of cement hydration. CNTs only improve mechanical properties by decreasing internal voids in the microstructure and the role of the nucleation seed that promotes C-S-H.

### 4.3. Analysis of Mechanical Properties

**Figure 7:**
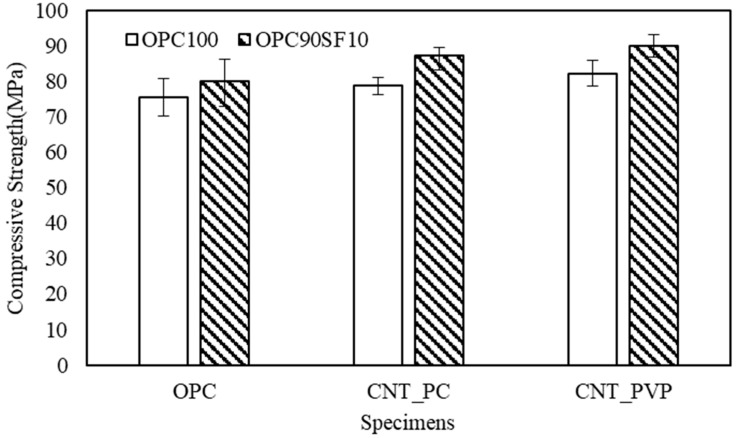
Compressive strength of cement composites.

**Figure 8:**
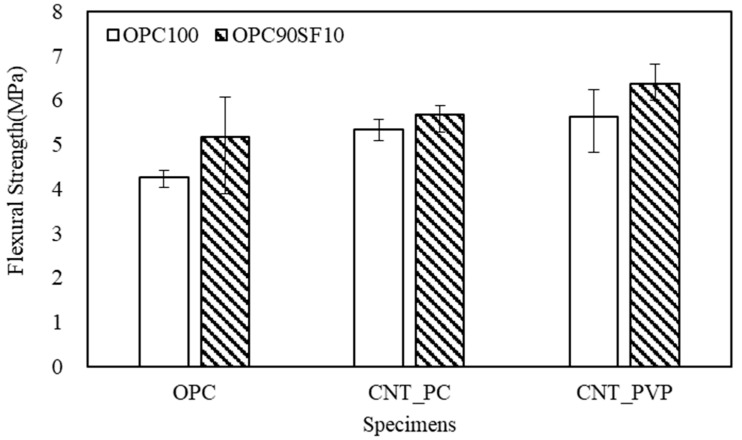
Flexural strength of cement composites.

To analyze the mechanical properties of the specimens, the compressive strength and flexural strength were measured based on the 28th day, and the results are shown in Figure 7 and Figure 8. The compressive strength of OPC was measured at 75.53 MPa, and with 10% replacement of silica fume, the compressive strength increased to 80.27 MPa. When using CNT aqueous dispersions prepared with PC-based surfactants, the compressive strength was 78.78 MPa, and with 10% replacement of silica fume, the compressive strength increased to 87.33 MPa. When using CNT aqueous dispersions prepared with PVP surfactants, the compressive strength was 82.20 MPa, and with a 10% replacement of silica fume, the compressive strength increased to 90.10 MPa.

When CNT aqueous dispersion was added, an increase in the strength was confirmed in all of the specimens regardless of the type of surfactant, and among the cement paste formulation with a mixing of CNTs, along with the use of PC-based surfactants, an improvement of 4.3% in the strength was confirmed compared to that of OPC, and by using PVP as a surfactant, the strength improved by 8.8%. The results showed that PVP was effective at obtaining a relatively high compressive strength. With the replacement of the silica fume at 10%, the compressive strength increased by approximately 6.2% compared to OPC, and when the CNT aqueous dispersion was added, a high rate of increase in the strength (approximately 9.6% to 10.8%) was confirmed regardless of the type of surfactant. The reason is believed to be that CNTs also contributed to the effect of silica fumes, causing a much denser microstructure of the voids [35].

Regarding the flexural strength, similar to the case with the compressive strength, when CNTs were mixed, the effect of strength enhancement was confirmed. In the case of OPC, the flexural strength was measured at 4.26 MPa, and with a 10% replacement of the silica fume, the flexural strength increased to 5.17 MPa. Among CNT aqueous dispersions, when using PC-based superplasticizer as a surfactant, the flexural strength was 5.33 MPa, and with a 10% replacement of silica fume, the flexural strength increased to 5.67 MPa. When using CNT aqueous dispersions prepared with PVP surfactants, the flexural strength was 5.62 MPa, and with a 10% replacement of the silica fume, the flexural strength increased to 6.37 MPa. In particular, when the CNT aqueous dispersion was mixed, the flexural strength showed a relatively high increase rate of approximately 25% to 32% compared to the increase rate of compressive strength.

The effect of silica fume replacement showed a relatively high increase rate of approximately 21% in OPC, but a low increase rate of approximately 7–13% with the mixing of the CNT aqueous dispersions. This result is opposite to that of the compressive strength, and an additional analysis should be performed to clarify. When comparing the mechanical properties of OPC–CNT composites, the PVP surfactant showed better strength, but PC-based surfactants also showed the effect of strength improvement compared to that of OPC. In particular, similar rates of increase in compressive strength and flexural strength were confirmed in the specimens without silica fume replacement, and because admixtures were not added to ensure flowability, the advantage of mixing CNT aqueous dispersions was confirmed in terms of its usability.

### 4.4. Results of SEM Analysis

SEM analysis was performed to analyze (1) the microstructural changes resulting from the addition of CNTs to the cement paste, and (2) to investigate the effect on CNT dispersibility and cement hydrate formation in the cement paste of two types of surfactants used in the preparation of OPC–CNT composites.

**Figure 9:**
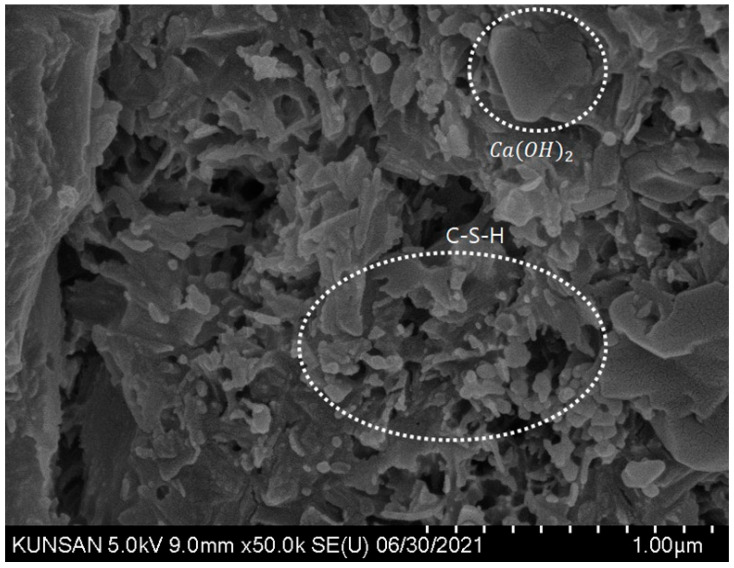
OPC SEM image of cement composites.

**Figure 10:**
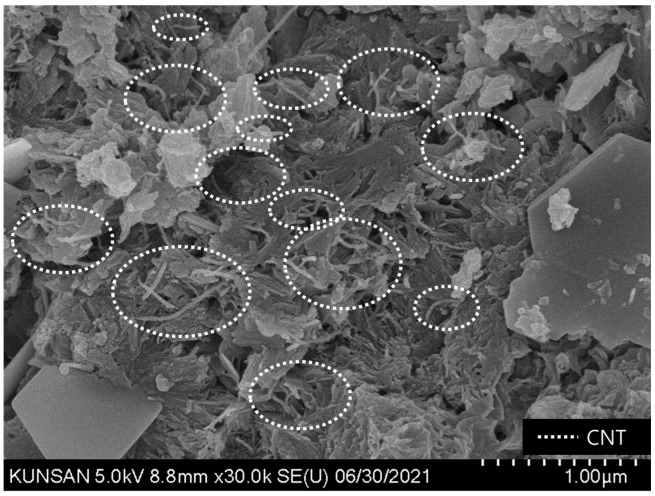
CNT_PVP SEM image of cement composites—well distributed.

**Figure 11:**
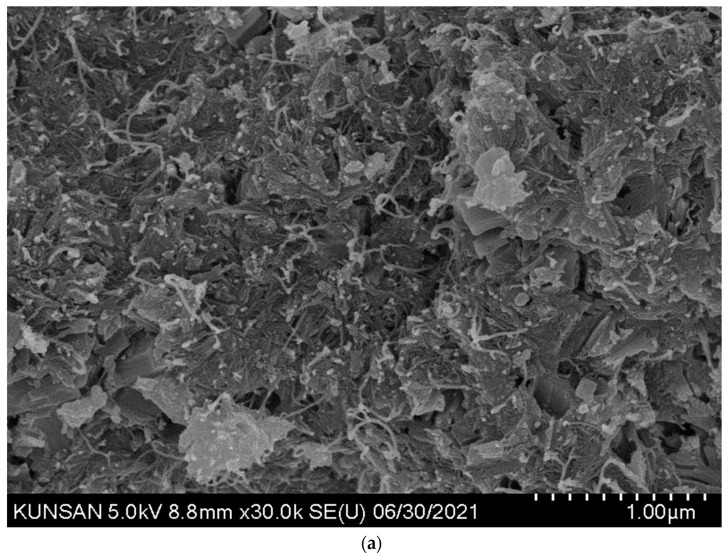

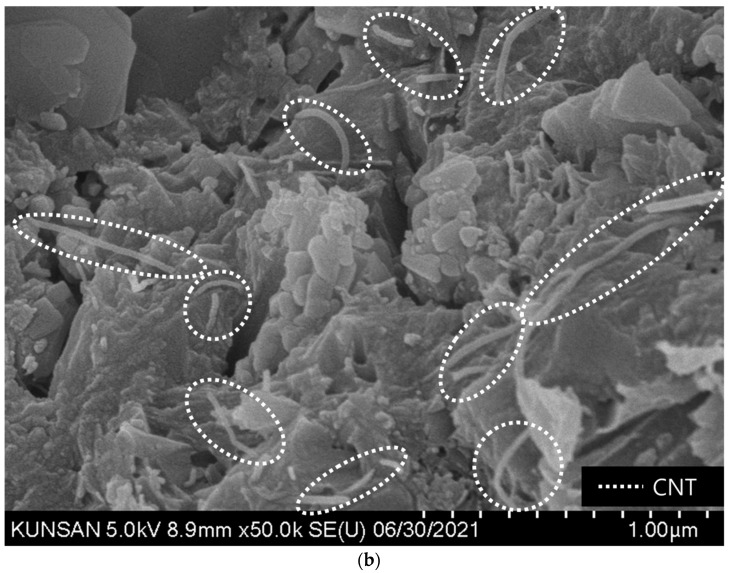
CNT_PC SEM image of cement composites—well distributed. (**a**) Low-magnification image; (**b**) high-magnification image.

**Figure 12:**
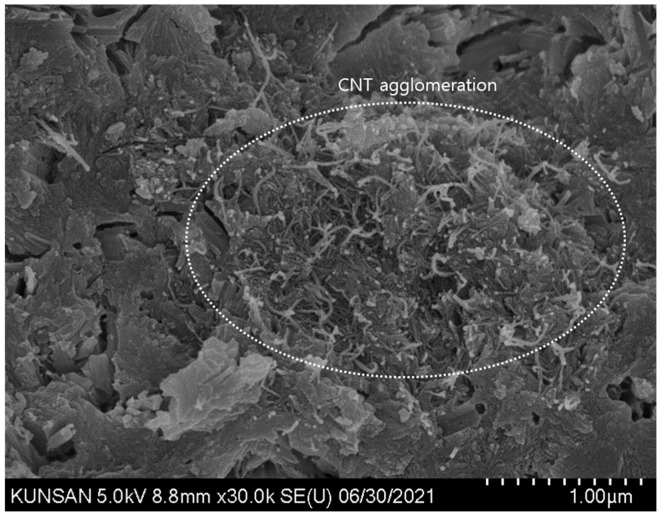
CNT_PC SEM image of cement composites—not well distributed.

For comparative analysis with the SEM images of the specimens mixed with CNT dispersions, images of the control OPC specimen were obtained and analyzed, and the formation of major hydration products such as C-S-H and Ca(OH)_2_ was confirmed (Figure 9). In the case of cement specimens with CNT aqueous dispersions, a thin and long CNT morphology can be seen in all SEM images regardless of the type of surfactant. In addition, by binding the C-S-H and CNT, the structure of the internal voids was filled, leading to a denser microstructure with voids (Figure 10 and Figure 11). When PVP-based surfactants were used, the SEM images confirmed that CNTs were evenly dispersed in the cement paste, and when PC-based surfactants were used, some aggregation of CNTs was observed, as shown in Figure 12. It can be seen that the CNT aqueous dispersion prepared with PVP surfactants showed a more effective dispersion of CNTs in the cement paste compared to the PC-based surfactants. However, in this study, even in the case involving the use of PC-based surfactants, a preparation method based on the dispersion technique optimized for PVP surfactant was used. Therefore, additional research is required from this perspective. That is, additional research is needed for the preparation of CNT aqueous dispersions with PC-based superplasticizer used in cement concrete; the results of this study demonstrated that PC-based surfactants for concrete have sufficient applicability for use in the preparation of CNT aqueous dispersions.

### 4.5. Results of BET Analysis

**Figure 13:**
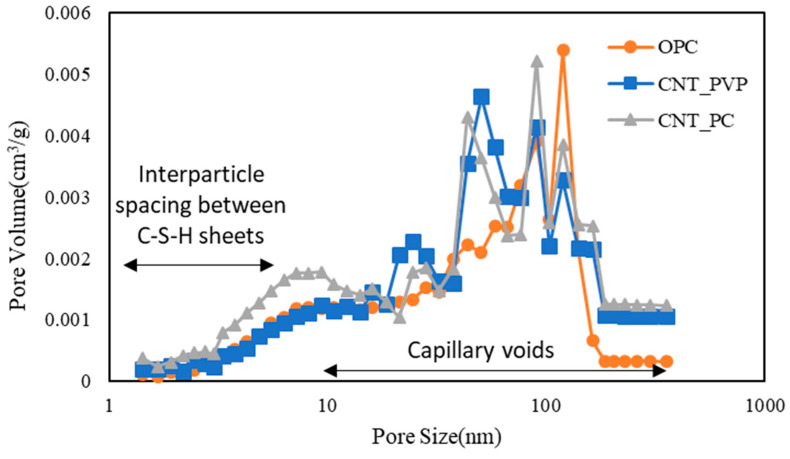
Pore volume with respect to pore size of cement composites (comparison between different CNT dispersions).

**Figure 14:**
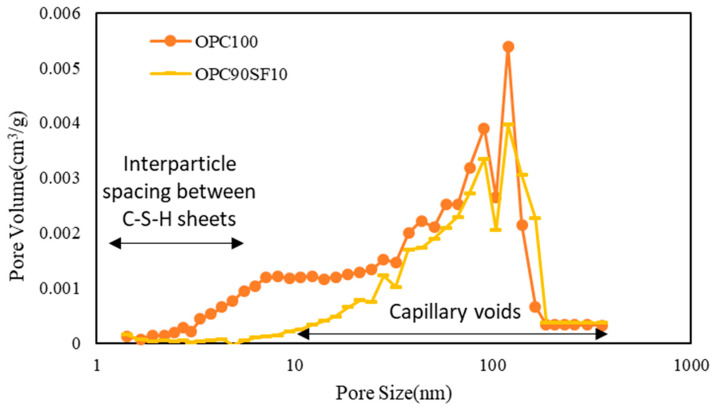
Pore volume with respect to pore size of cement composites.

**Figure 15:**
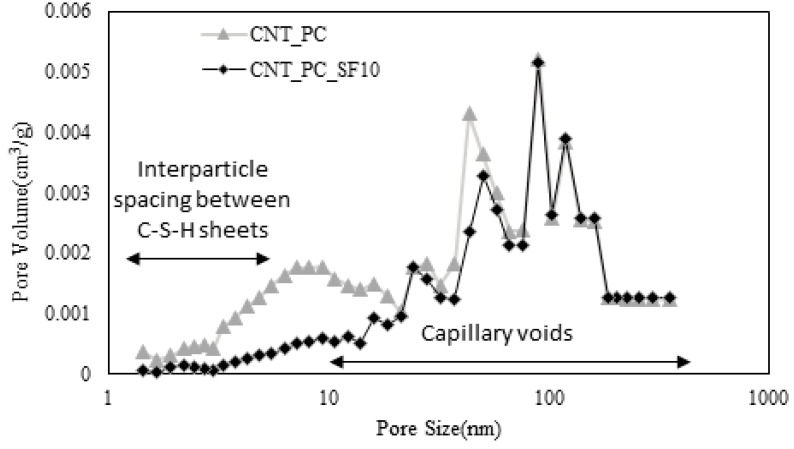
Pore volume with respect to pore size of cement–CNT composites.

**Figure 16:**
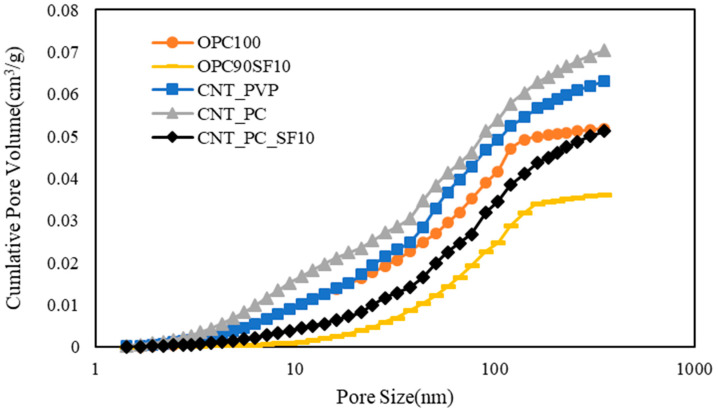
Cumulative pore volume with respect to pore size of cement composites.

The results of the BET analysis on the 28th day for the OPC–CNT composites microstructure are shown in Figure 13, Figure 14 and Figure 15, and the results of the total cumulative pore volume are presented in Figure 16. In the case of the OPC–CNT composites, as shown in Figure 13, micropores were observed between the C-S-H sheets, and most of the voids were measured as capillary voids. In the case of the paste mixed with CNT aqueous dispersions, there was a result of dispersing the pore volume per unit weight of capillary voids with a size of 100 nm. When PC-based surfactants were used for the preparation of CNT aqueous dispersions, there was a relative increase in the micropores between the C-S-H sheets. However, the overall results were similar to those of PVP surfactants.

Figure 14 shows the pore volume per unit weight according to the pore size with 10% of the admixture replacement with a silica fume. Owing to the effect of the silica fume, the volume of micropores between C-S-H sheets significantly decreased compared to that of 100% OPC, and most of the voids were measured as capillary voids. As shown in Figure 15, a similar result was observed in the case involving mixing the CNT aqueous dispersions prepared with PC-based superplasticizer. Therefore, the results confirmed that by mixing CNT aqueous dispersions, the effect of filling the micropores between C-S-H sheets was insignificant compared to the effect of the silica fume.

The cumulative pore volume per unit weight increased by approximately 21–35% when the CNT aqueous dispersion was added, as shown in Figure 16. This reflects the increase in mesopores owing to an increase in the amount of air introduced because of the addition of surfactants. Increasing mesopores may also cause deterioration in mechanical performance and durability. However, no mechanical performance degradation occurred.

It is considered that CNTs function as nucleation seeds to accelerate the initial cement hydration, increasing the specific surface area in which C-S-H promotes the initial cement hydration, which leads to a relatively greater pore volume compared to that of OPC. The dispersant used to disperse CNTs caused an air entrainment effect inside the cement paste, resulting in an increase in capillary pores. Therefore, the effect on the microstructure appears to be greater, due to the PC-based superplasticizer used as a dispersant, than the effect on CNTs’ function as nucleation seeds.

Silica fume is mixed with the filler effect. The internal voids between the C-S-H sheets are filled to reduce the total volume. The same effect was confirmed in the case involving the addition of CNT aqueous dispersions. Therefore, it is believed that the mechanical properties were enhanced, owing to the increase in strength because of the improved microstructure of the CNT.

CNT aqueous dispersions were mixed, and the pore volume was increased compared to that of the OPC, but the difference between the PC-based and PVP surfactants was not significant. Therefore, the PC-based superplasticizer would be sufficient for the preparation of CNT aqueous dispersions, and it was determined that examining the admixture ratio would be effective for improving the microstructure.

## 5. Conclusions

In this study, the mechanical properties and microstructure of cement paste mixed with CNT aqueous dispersions using PC-based surfactants were investigated, and their usability was evaluated. The results were organized as follows.

(1) Based on the results of the Vicat needle test for the measurement of the penetration resistance, by mixing CNT aqueous dispersions, the effect of reducing the initial setting time was observed, and the type of surfactant for CNT dispersions had an insignificant effect. After the initial setting, the setting rate gradually decreased, and the final setting time was measured at values similar to those of the OPC.
(2) Based on the TGA results, the weight loss curves of the specimens with CNT aqueous dispersions showed a similar result to that of the OPC specimen. This indicates that, when considering the result of the Vicat needle test, in the formation of hydrates, there is no chemical reaction between the CNT and cement, but CNTs only affected nucleation and physical bridging.
(3) From the analyses of the mechanical properties, when the CNT aqueous dispersion was added, the compressive strength increased to be 9.6–10.8% higher than that of OPC. A similar compressive strength was measured when PVP surfactants were used as compared to PC-based surfactants. The PVP surfactants wrapping the CNT are superior to the PC-based surfactants, which was also confirmed by the SEM image analysis. However, using PC-based surfactants, the strength improvement effect was confirmed to be similar, indicating a sufficient level of usability of the PC-based surfactants.
(4) From the results of measuring the flexural strength of CNT–cement composites, the strength improvement was confirmed, as is shown in the compressive strength test. The effect of mechanical property enhancement was higher in terms of the flexural strength compared to the effect of compressive strength improvement. Based on SEM image analysis, it was confirmed that CNTs, compared to the results of BET analysis, the space between the C-S-H hydrates, and capillary voids, were filled by silica fumes and CNTs, leading to a denser internal void structure, thereby enhancing the mechanical properties.
(5) Based on the results of analyzing the microstructure, when CNT aqueous dispersions were mixed, the pore volume per unit weight increased. Both of the CNT aqueous dispersions dispersed with PC-based surfactants, and PVP surfactants played a role in dispersing the size of capillary voids, which led to an improvement in the mechanical properties. Therefore, based on the findings of this study, the use of the PC-based surfactant as a CNT dispersant requires additional improvement, but it is considered to have sufficient potential for usability.

## Figures and Tables

**Table 1:** Characteristics of CNT.

Notation	Purity (%)	Diameter (nm)	Length (nm)	Specific Surface Area (m^2^/g)	True Density (g/cm^3^)
CNT 6A	>97.5	5–7	50–150	200–300	2.22

**Table 2:** Cement paste mix proportion.

Specimens	W/B (%)	CNT (%)	Binder (%)		CS/B (%)	Surfactant (type)	Superplasticizer (%)
			C	SF			
OPC	30	0	100	0	0		-
SF10			90	10			-
OPC_PC			100	0			0.05
CNT_PVP	30	0.1	100	0	10	PVP	-
CNT_PVP_SF10			90	10			
CNT_PC			100	0		PC	
CNT_PC_SF10			90	10			

W: Water; B: binder; C: Ordinary Portland Cement; SF: silica Fume; CS: CNT 1% Solution; PVP: polyvinyl pyrrolidone; PC: (polycarboxilic)-based superplasticizer.

**Table 3:** Chemical properties of binders.

Composition %	Cement (OPC)	Silica Fume
CaO	61.33	0.38
Al_2_O_3_	6.40	0.25
SiO_2_	21.01	96.00
Fe_2_O_3_	3.12	0.12
MgO	3.02	0.10
SO_3_	2.30	-
Specific surface (cm^2^/g)	3413	200,000
Loss ignition (%)	1.40	1.50
Density (g/cm^3^)	3.15	2.10

## Data Availability

Not applicable.
